# The Morphology of a Kinematically Aligned Distal Femoral Osteotomy Is Different from That Obtained with Mechanical Alignment and Could Have Implications for the Design of Total Knee Arthroplasty

**DOI:** 10.3390/jpm12030422

**Published:** 2022-03-08

**Authors:** Quan-Hu Shen, Ji-Woong Baik, Ye-Yeon Won

**Affiliations:** 1 Department of Orthopaedic Surgery, Ajou University College of Medicine, Ajou Medical Center, Suwon 16499, Korea; shenquanhu@gmail.com (Q.-H.S.); bjy1991@naver.com (J.-W.B.); 2 Department of Orthopeadic Surgery, First People’s Hospital of Suqian City, Suqian 223800, China

**Keywords:** total knee arthroplasty, kinematic alignment, mechanical alignment, morphotype

## Abstract

Background: Kinematically aligned total knee arthroplasty (KA-TKA) may lead to a different pattern of osteotomy from mechanically aligned total knee arthroplasty (MA-TKA). This paper aims to analyze the effects of KA and MA on the morphology of the distal femoral osteotomy surface. Methods: Computed tomography scans of 80 TKA candidates were reconstructed into 3D models. The measurement of bone morphology was performed after the distal femur cut according to two different alignment techniques. The aspect ratio, trapezoidicity ratio, and asymmetry ratio of the distal femur were assessed. Results: The aspect ratio and the asymmetry ratio in the KA group was significantly lower than that in the MA group in the general population (*p* < 0.001). The trapezoidicity ratio in the KA group was significantly higher than that in the MA group in the general population (*p* < 0.001). Conclusions: It was found that KA-TKA and MA-TKA presented different morphologies of the distal femoral osteotomy surface, and this difference was also influenced by gender. The surgery pattern of KA-TKA and MA-TKA and gender should be considered when surgeons choose femoral prostheses.

## 1. Introduction

Total knee arthroplasty (TKA) has been proven successful in improving knee pain and functions and the quality of life of patients with advanced knee arthritis [1]. The choice of prosthesis that matches the bone surface morphology after cutting is of high importance, otherwise mismatching may influence pain, function, and flexion after TKA [2,3]. It has been reported that bone-implant mismatch can occur in TKA due to variation in the aspect ratio (ML/AP; ML = medial-lateral width, AP = anteroposterior length) and the asymmetry ratio (LAP/MAP; LAP = lateral anteroposterior, MAP = medial anteroposterior) [4]. Marmor et al. reported that over-voluming occurred in 24% of TKA pre-operative planning [5]. In the past decade, studies on distal femur morphology have primarily focused on the aspect ratio of the distal femur [6,7,8,9,10]. Based on current findings, manufacturers tend to produce narrow femoral prosthesis to improve match with bone surface morphology.

Mahfouz et al. described the complex variation of femur morphology, considering it to be too simplistic to divide distal femur morphology into wide and narrow [2]. Trapezoidicity is a recently proposed measure for describing distal femur morphology. Distal femurs are reported to be more trapezoidal than the common femoral implants in Caucasians [11]. Although some researchers have reported ethnic and gender differences in distal femur morphology [11,12,13], there are no such differences in prostheses used for TKA.

Mechanical alignment (MA) TKA is often considered the gold standard alignment technique for TKA. MA-TKA aims to restore mechanical alignment and achieve a joint line perpendicular to the mechanical axis of the lower extremities. In recent studies, kinematically aligned (KA) TKA has been found to achieve good outcomes in TKA [14,15,16]. KA-TKA is differentiated from MA-TKA in that the former aims to restore the thickness of the bones and cartilages removed, with the external rotation of the femur set at 0° to the anatomical joint line [17]. However, to the best of our knowledge, no study has compared the morphology of the distal femoral osteotomy surface using KA-TKA and MA-TKA techniques.

Therefore, the purpose of this study was to compare the aspect ratio, trapezoidicity ratio, and asymmetry ratio of the distal femur in KA and MA groups. We hypothesized that the morphology of a kinematically aligned distal femoral osteotomy would be different from that obtained with mechanical alignment.

## 2. Materials and Methods

The present study was approved by the institutional review board of our institution. Measurement was performed on the preoperative CT scan images of the lower limbs in 86 patients scheduled for primary TKA from 2014 to 2015. There were 31 males and 55 females. Six patients were excluded due to a history of surgery or trauma, or unclear CT images caused by artifacts related to metal or contrast agents. Finally, 30 males and 50 females were included, who were aged 72 ± 2.6 years old on average, with an average body mass index of 26.8 ± 3.5 kg/m^2^ (Table 1). All the patients included had knee osteoarthritis with varus deformity before surgery.

The CT images were acquired in a supine position using a 64-slice multi-detector CT unit (Siemens Sensation, Munich, Germany) for all patients. The CT scan was performed in full knee extension, with two legs immobilized in neutral rotation.

CT scans were imported to special medical imaging software (Xelis software, version 1.0.2.2; INFINITT, Seoul, Korea) to create three-dimensional (3D) reconstruction models and simulate femoral cutting.

### 2.1. Virtual Surgery

**Figure 1:**
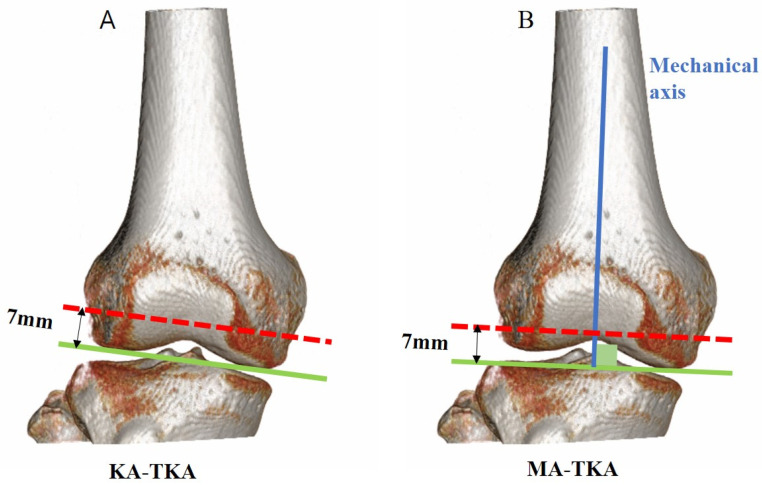
(**A**) Distal femoral cutting of kinematically aligned total knee arthroplasty (KA-TKA). (**B**) Distal femoral cutting of mechanically aligned total knee arthroplasty (MA-TKA). The cutting thickness was 7 mm.

**Figure 2:**
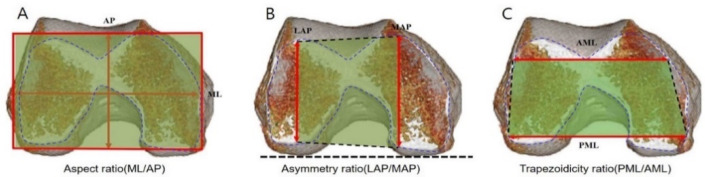
Three geometric ratios describe the morphology of bone after distal femur cutting: (**A**) aspect ratio (ML/AP), (**B**) asymmetry ratio (LAP/MAP), and (**C**) trapezoidicity ratio (PML/AML). The maximum medial AP perpendicular to the line connecting the medial condyle and the posterior margin of the medial and lateral condyles was taken as medial anteroposterior (MAP). The maximum lateral AP was lateral anteroposterior (LAP). The larger of the two values was set to the AP value. The posterior medial-lateral (PML) width was the posterior femoral condylar cutting width and the anterior medial-lateral (AML) width was measured at 75% AP.

**Figure 3:**
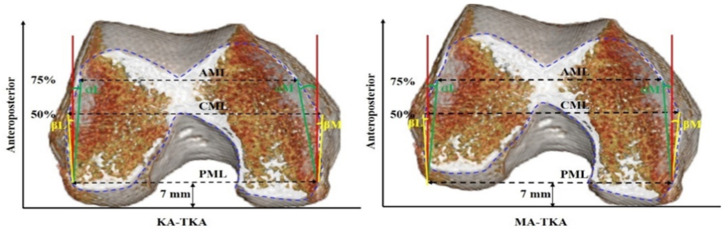
Transverse CT slice at the level of the distal cutting of the femur, indicating the femoral cortical contours (blue). The posterior medial-lateral (PML) width was the posterior femoral condylar cutting width, the central medial-lateral (CML) width was measured at 50% AP, and the anterior medial-lateral (AML) width was measured at 75% AP. Lateral narrowing: αL and βL; medial narrowing: αM and βM.

KA-TKA was performed as follows: The distal femoral cutting was simulated on a plane parallel to the line facing the distal surfaces of both condyles (Figure 1A). The thickness of the distal femoral cutting was 7 mm considering a mean articular cartilage thickness of 2 mm. In other words, 9 mm thickness resected was to substitute the corresponding region of the femoral component. The maximum medial AP perpendicular to the line connecting the medial condyle and the posterior margin of the medial and lateral condyles was taken as medial anteroposterior (MAP). The maximum lateral AP was lateral anteroposterior (LAP). The larger of the two values was set to the AP value (Figure 2). The medial-lateral widths were measured at three positions: from the outmost points of the medial and lateral posterior condyles, the posterior medial-lateral (PML) width was measured at 7 mm anteriorly, the central medial-lateral (CML) width was measured at 50% AP, and the anterior medial-lateral (AML) width was measured at 75% AP (Figure 3). Narrowing angles were measured between the line perpendicular to the PML line and the cortex at 75% (angle α or ‘anterior narrowing angle’) and at 50% (angle β or ‘central narrowing angle’) of the AP. Angles were measured both on the medial (αM and βM) and on the lateral side (αL and βL) (Figure 3).

MA-TKA was performed as follows: The distal femoral cutting was simulated on a plane perpendicular to the femoral mechanical axis (Figure 1B). The thickness of the distal femoral cutting was 7 mm. The maximum medial AP perpendicular to the line connecting the medial condyle and the posterior margin of the lateral condyle was taken as MAP. The maximum lateral AP was LAP. The larger of the two values was set to the AP value (Figure 2). The medial-lateral widths were measured at three positions: parallel with surgical epicondylar axis and from the outmost point of the posterior femoral condyle, PML was measured at 7 mm anteriorly, CML was measured at 50% AP, and AML was measured at 75% AP. Narrowing angles were measured between the line perpendicular to the PML line and the cortex at 75% (angle α or ‘anterior narrowing angle’) and at 50% (angle ß or ‘central narrowing angle’) of the AP. Angles were measured both on the medial (αM and βM) and on the lateral side (αL and βL) (Figure 3).

This simulation has previously been validated in research by Kim et al. [18].

The aspect ratio (ML/AP), asymmetry ratio (LAP/MAP), and trapezoidicity ratio (PML/AML) of the distal femur were assessed (Figure 2). For each ratio, we classified the shape relative to the median value: femurs were considered wide if the aspect ratio was above the median; femurs were considered narrow if the aspect ratio was below the median; femurs were trapezoidal if the trapezoidicity ratio was above the median; femurs were rectangular if the trapezoidicity ratio was below the median.

### 2.2. Statistical Analysis

Statistical analysis was performed using the IBM SPSS Statistics software package (IBM SPSS Statistics 21, SPSS IBM, NY, USA). Descriptive statistics were used to summarize the data. Data were presented as mean ± SD. Shapiro–Wilk tests were used to assess the normality of distributions. An independent sample *t* test was used to test statistical significance. *p* values < 0.05 were considered statistically significant.

All the measurements on the analysis were made by one orthopedic surgeon. Another orthopedic surgeon measured 30 randomly selected patients to assess interobserver reliability of measurements. The intraclass correlation coefficient of interobserver reliability was 0.88 which was indicative of excellent agreement. The intraobserver agreement rate was 0.94.

## 3. Results

The aspect ratio (ML/AP) in the KA group was significantly lower than that in the MA group in the general population (*p* < 0.001, Table 2), males (*p* < 0.001, Table 3), and females (*p* < 0.001, Table 4).

The asymmetry ratio (LAP/MAP) in the KA group was significantly lower than that in the MA group in the general population (*p* < 0.001, Table 2), males (*p* = 0.01, Table 3), and females (*p* < 0.001, Table 4).

The trapezoidicity ratio (PML/AML) in the KA group was significantly higher than that in the MA group in the general population (*p* < 0.001, Table 2), males (*p* < 0.001, Table 3), and females (*p* < 0.001, Table 4).

Overall αL was significantly smaller in the KA group than in the MA group (*p* < 0.001). Overall βL was significantly smaller in the KA group than in the MA group (*p* = 0.002). There was no significant difference in overall αM between KA and MA groups (*p* = 0.189). Overall βM was significantly larger in the KA group than in the MA group (*p* < 0.001) (Table 2).

All of the indicators above of females were lower than those of males in the KA group. In the MA group, there was no gender difference in LAP/MAP and βM. All of the remaining indicators were lower in females than in males (Table 5).

Based on the median, distal femur morphology was divided into wide/narrow or rectangle/trapezoid. There were 24 (30%) narrow trapezoidal femurs, 16 (20%) narrow rectangular femurs, 16 (20%) wide trapezoidal femurs, and 24 (30%) wide rectangular femurs in the KA group. In the MA group, there were 20 (25%) femurs of each morphology. Narrow trapezoidal femurs and wide rectangular femurs were more common in the KA group.

## 4. Discussion

The most important finding of the present study was that MA-TKA and KA-TKA resulted in significantly different distal femur morphology after cutting and this difference was also influenced by gender.

The distal femur morphology after cutting was related to gender in both techniques, which supported the common belief that the aspect ratio is lower in females [11,12,19]. Li et al. reported that the aspect ratio of females and males was 1.15 ± 0.09 vs. 1.18 ± 0.12 (*p* = 0.005) in the Chinese population [12]. Another report found that the aspect ratio of females and males was 1.14 ± 0.06 vs. 1.19 ± 0.08 (*p* < 0.001) [11]. Concerning the symmetry ratio, it was reported that females were more asymmetric, with a symmetry ratio 1.02 ± 0.05 compared to 1.02 ± 0.05 of males [12]. However, another study found that there was no significant difference in the symmetry ratio between females and males [11]. Therefore, it is undesirable to use MA-TKA implants for KA-TKA. A special implant design for KA-TKA is needed. However, even asymmetric implants for KA-TKA might be used with MA-TKA osteotomy, such as the Smith and Nephew Journey II series [20]; thus, KA-TKAs do not necessarily involve distal femoral osteotomy parallel to the articular surface.

Bonnin et al. compared the geometric shape of the distal femur against existing ready-made prostheses. They pointed out that the trapezoidicity ratio of most femoral prostheses was low compared with the distal femurs, resulting in insufficient coverage by the prosthesis [11]. Some believe that distal femur cutting with external rotation of the femur set at 0° may affect the trapezoidicity ratio. However, according to our study, the two alignment techniques did not result in a significant difference in the trapezoidicity ratio. Mahfouz et al. reported an ethnic difference but no gender difference in the trapezoidicity ratio [2]. We found no gender difference in the trapezoidicity ratio between the two alignment techniques (Table 5). The most common distal femur morphologies in KA-TKA were narrow trapezoid and wide rectangle. The numbers of femurs with four distal femur morphologies were equal in the MA-TKA group.

The lateral narrowing angle was smaller in KA-TKA than in MA-TKA, and the medial narrowing angle was smaller in the former than in the latter. The medial narrowing angle in MA-TKA was almost twice that of the lateral narrowing angle. In KA-TKA, the medial narrowing angle was almost three times that of the lateral narrowing angle (Table 2). This result agrees with the findings of Bonnin et al. [11].

The actual distal femur morphology is much more complex than a simple division into narrow and wide. Therefore, existing ready-made distal femoral prostheses can hardly cover the bone surface after cutting. Postoperative residual pain and rigidity are related to protrusion of the prosthesis and excessive size of the femoral components [5]. Implants not matching the bone surface after cutting may lead to ligament balance, patellar dislocation, and soft tissue impingement [11]. All of these conditions have an adverse impact on functional prognosis. Our study presented the effect of alignment patterns of KA and MA on the aspect ratio and symmetry ratio of the distal femoral osteotomy surface in TKA. These findings should provide insights to manufacturers with respect to the design of femoral prosthesis, and to clinicians regarding the selection of different types of prosthesis considering the alignment patterns of KA or MA and gender during TKA.

The major limitation of this study is that all of our observed subjects were Korean. Since ethnic differences are assumed to occur in distal femur morphology, it remains unclear whether our findings are also suitable for Caucasians or Africans. Secondly, the sample size was small in this study. However, a significant difference was observed between the two alignment techniques in TKA. We are still uncertain what clinical implications arise from such a difference. Lastly, we only performed the correlation analysis in preoperative varus hip-knee-ankle angle not including valgus deformity. As most TKA candidates have slight varus deformity, these results cannot be applied to patients with valgus or dysplastic femoral deformity.

## 5. Conclusions

It was found that KA-TKA and MA-TKA presented different morphologies of the distal femoral osteotomy surface, and that this difference was also influenced by gender. These findings provide insights for the development of individualized TKA considering differences in the morphology of the distal femoral osteotomy surface caused by different alignment patterns of KA and MA.

## Figures and Tables

**Table 1:** Patient demographic and BMI data.

	Total (*n* = 80)	Females (*n* = 50)	Males (*n* = 30)
	Mean ± SD (range)	Mean ± SD (range)	Mean ± SD (range)
Age (year)	72 ± 2.6 (52–77)	73 ± 3.7 (61–80)	70 ± 2.5 (52–79)
BMI (kg/m^2^)	26.8 ± 3.5 (19.3–33.5)	30.2 ± 5.6 (19.3–33.5)	25.5 ± 3.2 (20.4–28.4)

SD: standard deviation; BMI: body mass index.

**Table 2:** Distal femoral dimensions.

	KA-TKA (*n* = 80)	MA -TKA (*n* = 80)	
	Mean ± SD (range)	Mean ± SD (range)	*p* value
MAP (mm)	49.5 ± 2.5 (43.1–54.8)	48.8 ± 3.9 (38.8–59.0)	0.141
LAP (mm)	51.9 ± 3.0 (45.5–60.5)	46.2 ± 4.2 (37.6–55.9)	0.000
AML (mm)	59.3 ± 4.5 (50.4–70.7)	59.1 ± 4.8 (49.8–70.4)	0.802
CML (mm)	65.5 ± 4.8 (56.8–77.6)	66.3 ± 5.5 (57.3–77.3)	0.319
PML (mm)	67.8 ± 5.7 (57.1–79.9)	69.7 ± 6.3 (58.4–82.4)	0.042
ML/AP	1.3 ± 0.1 (1.2–1.5)	1.4 ± 0.1 (1.3–1.6)	0.000
PML/AML	1.1 ± 0.1(1.0–1.3)	1.2 ± 0.1 (1.1–1.3)	0.000
LAP/MAP	1.0 ± 0.0 (1.0–1.2)	0.9 ± 0.0 (0.8–1.1)	0.000
αL (°)	4.0 ± 3.1 (−5.2–10.3)	8.8 ± 4.1 (−2.9–20.2)	0.000
βL (°)	1.4 ± 5.8 (−14.5–11.1)	4.2 ± 5.5 (−9.1–16.1)	0.002
αM (°)	11.5 ± 4.3 (2.2–23.0)	12.3 ± 5.0 (4.0–21.7)	0.189
βM (°)	4.2 ± 5.3 (−10.6–14.9)	11.6 ± 5.0 (−7.4–23.9)	0.000

KA-TKA: kinematically aligned total knee arthroplasty; MA-TKA: mechanically aligned total knee arthroplasty; SD: standard deviation; MAP: medial anteroposterior length; LAP: lateral anteroposterior length; AML: anterior medial-lateral width; CML: central medial-lateral width; PML: posterior medial-lateral width; ML/AP: aspect ratio; LAP/MAP: asymmetry ratio; PML/AML: trapezoidicity ratio; lateral narrowing: αL and βL; medial narrowing: αM and βM.

**Table 3:** Comparison of male distal femoral dimensions by two types of surgery.

	KA-TKA (*n* = 30)	MA-TKA (*n* = 30)	
	Mean ± SD (range)	Mean ± SD (range)	*p* value
MAP (mm)	50.6 ± 2.5 (45.0–54.8)	51.6 ± 3.5 (43.8–59.0)	0.190
LAP (mm)	53.9 ± 3.0 (48.7–60.5)	48.5 ± 4.2 (41.5–55.9)	0.000
AML (mm)	63.2 ± 3.8 (56.8–70.7)	63.6 ± 3.6 (56.8–70.4)	0.666
CML (mm)	70.2 ± 3.3 (63.9–77.6)	72.1 ± 3.4 (65.8–77.3)	0.028
PML (mm)	73.7 ± 3.6 (63.3–79.9)	76.3 ± 4.0 (69.3–82.4)	0.011
ML/AP	1.4 ± 0.1 (1.2–1.5)	1.5 ± 0.1 (1.3–1.6)	0.000
PML/AML	1.2 ± 0.6 (1.1–1.3)	1.2 ± 0.5 (1.1–1.3)	0.010
LAP/MAP	1.1 ± 0.0 (1.0–1.2)	0.9 ± 0.1 (0.8–1.0)	0.000
αL (°)	5.4 ± 2.8 (−0.6–10.3)	11.0 ± 3.7 (3.4–20.2)	0.000
βL (°)	3.3 ± 5.1 (−7.1–11.1)	5.9 ± 5.2 (−4.6–16.1)	0.054
αM (°)	13.2 ± 4.5 (2.2–23.0)	13.4 ± 3.9 (4.0–21.7)	0.807
βM (°)	6.3 ± 4.7 (−3.6–14.9)	11.6 ± 5.8 (−7.4–23.9)	0.000

KA-TKA: kinematically aligned total knee arthroplasty; MA-TKA: mechanically aligned total knee arthroplasty; SD: standard deviation; MAP: medial anteroposterior length; LAP: lateral anteroposterior length; AML: anterior medial-lateral width; CML: central medial-lateral width; PML: posterior medial-lateral width; ML/AP: aspect ratio; LAP/MAP: asymmetry ratio; PML/AML: trapezoidicity ratio; lateral narrowing: αL and βL; medial narrowing: αM and βM.

**Table 4:** Comparison of female distal femoral dimensions by two types of surgery.

	KA-TKA (*n* = 50)	MA-TKA (*n* = 50)	
	Mean ± SD (range)	Mean ± SD (range)	*p* value
MAP (mm)	48.9 ± 2.4 (43.1–54.6)	47.1 ± 3.0 (38.8–53.7)	0.001
LAP (mm)	50.7 ± 2.3 (45.5–57.9)	44.8 ± 3.6 (37.6–54.1)	0.000
AML (mm)	56.9 ± 3.1 (50.4–64.9)	56.4 ± 3.2 (49.8–65.9)	0.380
CML (mm)	62.6 ± 3.1 (56.8–69.2)	62.8 ± 3.1 (57.3–69.3)	0.808
PML (mm)	64.2 ± 3.3 (57.1–72.2)	65.8 ± 3.5 (58.4–75.0)	0.024
ML/AP	1.3 ± 0.1 (1.2–1.4)	1.4 ± 0.1 (1.3–1.6)	0.000
PML/AML	1.1 ± 0.0 (1.0–1.3)	1.2 ± 0.1 (1.1–1.3)	0.000
LAP/MAP	1.0 ± 0.0 (1.0–1.1)	0.9 ± 0.0 (0.8–1.1)	0.000
αL (°)	3.2 ± 3.0 (−5.2–9.5)	7.6 ± 4.0 (−2.9–16.6)	0.000
βL (°)	0.2 ± 5.9 (−14.5–10.9)	3.2 ± 5.4 (−9.1–13.5)	0.010
αM (°)	10.5 ± 3.9 (2.8–17.9)	11.7 ± 3.3 (4.3–18.7)	0.109
βM (°)	2.9 ± 5.3 (−10.6–12.2)	11.5 ± 4.5 (−2.7–20.9)	0.000

KA-TKA: kinematically aligned total knee arthroplasty; MA-TKA: mechanically aligned total knee arthroplasty; SD: standard deviation; MAP: medial anteroposterior length; LAP: lateral anteroposterior length; AML: anterior medial-lateral width; CML: central medial-lateral width; PML: posterior medial-lateral width; ML/AP: aspect ratio; LAP/MAP: asymmetry ratio; PML/AML: trapezoidicity ratio; lateral narrowing: αL and βL; medial narrowing: αM and βM.

**Table 5:** Comparison of distal femoral dimensions between males and females who underwent KA-TKA and MA-TKA.

	KA-TKA (Mean ± SD)			MA-TKA (Mean ± SD)		
	Females	Males	*p* Value	Females	Males	*p* Value
MAP (mm)	48.9 ± 2.4	50.6 ± 2.5	0.004	47.1 ± 3.0	51.6 ± 3.5	0.000
LAP (mm)	50.7 ± 2.3	53.9 ± 3.0	0.000	44.8 ± 3.6	48.5 ± 4.2	0.000
AML (mm)	56.9 ± 3.1	63.2 ± 3.8	0.000	56.4 ± 3.2	63.6 ± 3.6	0.000
CML (mm)	62.6 ± 3.1	70.2 ± 3.3	0.000	62.8 ± 3.1	72.1 ± 3.4	0.000
PML (mm)	64.2 ± 3.3	73.7 ± 3.6	0.000	65.8 ± 3.5	76.3 ± 4.0	0.000
ML/AP	1.3 ± 0.1	1.4 ± 0.1	0.000	1.4 ± 0.1	1.5 ± 0.1	0.000
PML/AML	1.1 ± 0.0	1.2 ± 0.6	0.001	1.2 ± 0.1	1.2 ± 0.5	0.004
LAP/MAP	1.0 ± 0.0	1.1 ± 0.0	0.001	0.9 ± 0.0	0.9 ± 0.1	0.263
αL (°)	3.2 ± 3.0	5.4 ± 2.8	0.002	7.6 ± 4.0	11.0 ± 3.7	0.000
βL (°)	0.2 ± 5.9	3.3 ± 5.1	0.021	3.2 ±5.4	5.9 ± 5.2	0.029
αM (°)	10.5 ± 3.9	13.2 ± 4.5	0.007	11.7 ± 3.3	13.4 ± 3.9	0.031
βM (°)	2.9 ± 5.3	6.3 ± 4.7	0.004	11.5 ± 4.5	11.6 ± 5.8	0.942

KA-TKA: kinematically aligned total knee arthroplasty; MA-TKA: mechanically aligned total knee arthroplasty; SD: standard deviation; MAP: medial anteroposterior length; LAP: lateral anteroposterior length; AML: anterior medial-lateral width; CML: central medial-lateral width; PML: posterior medial-lateral width; ML/AP: aspect ratio; LAP/MAP: asymmetry ratio; PML/AML: trapezoidicity ratio; lateral narrowing: αL and βL; medial narrowing: αM and βM.

## Data Availability

The datasets before and after analysis in this study are available from the corresponding author on reasonable request.
